# Structural evolution of GeMn/Ge superlattices grown by molecular beam epitaxy under different growth conditions

**DOI:** 10.1186/1556-276X-6-624

**Published:** 2011-12-12

**Authors:** Ya Wang, Zhiming Liao, Hongyi Xu, Faxian Xiu, Xufeng Kou, Yong Wang, Kang L Wang, John Drennan, Jin Zou

**Affiliations:** 1 Division of Materials, The University of Queensland, Brisbane, QLD 4072, Australia; 2 Department of Electrical and Computer Engineering, Iowa State University, Ames, IA, 50011, USA; 3 Department of Materials Science and Engineering, Zhejiang University, Hangzhou, 310027, China; 4 Department of Electrical Engineering, University of California at Los Angeles, CA, 90095, USA; 5 Centre for Microscopy and Microanalysis, The University of Queensland, Brisbane, QLD 4072, Australia

**Keywords:** ferromagnetic semiconductor, transmission electron microscopy, magnetic precipitation, molecular beam epitaxy

## Abstract

GeMn/Ge epitaxial 'superlattices' grown by molecular beam epitaxy with different growth conditions have been systematically investigated by transmission electron microscopy. It is revealed that periodic arrays of GeMn nanodots can be formed on Ge and GaAs substrates at low temperature (approximately 70°C) due to the matched lattice constants of Ge (5.656 Å) and GaAs (5.653 Å), while a periodic Ge/GeMn superlattice grown on Si showed disordered GeMn nanodots with a large amount of stacking faults, which can be explained by the fact that Ge and Si have a large lattice mismatch. Moreover, by varying growth conditions, the GeMn/Ge superlattices can be manipulated from having disordered GeMn nanodots to ordered coherent nanodots and then to ordered nanocolumns.

**PACS**: 75.50.Pp; 61.72.-y; 66.30.Pa; 68.37.L.

## Introduction

Since their discovery in the early 2000s [1,2], Mn-doped Ge-diluted magnetic semiconductors [DMS] have been extensively investigated due to their good compatibility with mainstream Si technology [3-14]. In order to obtain room temperature ferromagnetism, enormous efforts were devoted to the growth of high-quality defect-free GeMn DMS. However, in most cases, Mn-rich precipitates (e.g., Ge_3_Mn_5 _[4,6] and Ge_2_Mn_5 _[15]) tend to form during growth due to the low Mn solubility in Ge. It has also been found that the thickness of GeMn thin films plays a critical role in the formation of Mn-rich precipitates, and secondary precipitates are usually easier to nucleate in thicker thin films due to the active Mn diffusion [15]. Therefore, it is desirable to grow thinner films to avoid Mn-rich precipitates. Using this strategy, we previously fabricated precipitate-free Ge_0.95_Mn_0.05 _quantum dots with Curie temperature up to 400 K [7] and demonstrated electrical-controlled ferromagnetism. On the other hand, for practical applications, it is desirable to control the distribution of Mn and to avoid the formation of Mn-rich secondary phases. By employing a 'superlattice' method, we successfully obtained ordered GeMn nanodot arrays [16]. These nanodot arrays exhibit unique magnetic properties and show promising applications in spintronic devices. However, the effects of substrate, Mn concentration, and growth temperature on the behavior of the GeMn nanodots are not yet explored although it is critical to fundamentally understand the structural evolution of such ordered nanodots.

In this study, by transmission electron microscopy [TEM], we investigated the effect of substrate, GeMn/Ge thickness, Mn concentration, and growth temperature on the structure of GeMn nanodots grown by molecular beam epitaxy [MBE]. We observed a structural change from being disordered GeMn nanodots to ordered nanodots and then to ordered nanocolumns by varying the growth conditions. The reason behind this phenomenon is also discussed.

## Experimental details

Following a well-established growth approach [8,16], ten periods of GeMn/Ge superlattice were grown on various substrates (Si, Ge, and GaAs) at different temperatures (from room temperature to 150°C) by a PerkinElmer MBE (SVT Associates, formerly Perkin-Elmer, Physical Electronics Division, Eden Prairie, MN, USA), and the growth details are summarized in Table 1. By adjusting the Mn cell temperature, the Mn concentration of the GeMn layer can be changed. For example, when the Mn's cell temperature was set as 900°C, the nominal Mn concentration in the grown GeMn layer is approximately 12%. In Table 1, the nominal Mn concentrations in different GeMn layers were adjusted by the Mn's cell temperature. During the growth, reflection high-energy electron diffraction technique was applied to monitor the surface of the grown thin films. The detailed growth information can be found in the study of Wang et al. [16]. The grown thin films were then characterized by various TEM techniques on a Philips Tecnai F20 TEM (Philips Co., Holland, The Netherlands) operating at 200 kV.

**Table 1:** Sample details

	Sample code											
	S1	S2	S3	S4	S5	S6	S7	S8	S9	S10	S11	S12
Substrate	Ge	GaAs	Si	Ge	Ge	Ge	Ge	Ge	Ge	Ge	Ge	Ge
*T*_sub _(°C)	70	70	70	70	70	70	70	70	70	27	110	150
Mn (%)	12	12	12	12	12	7	8.5	10	14	12	12	12
Ge (nm)	11	11	11	11	4.6	11	11	11	11	11	11	11
GeMn (nm)	3	3	3	1.2	3	3	3	3	3	3	3	3

## Results and discussions

### The effect of substrates

**Figure 1:**
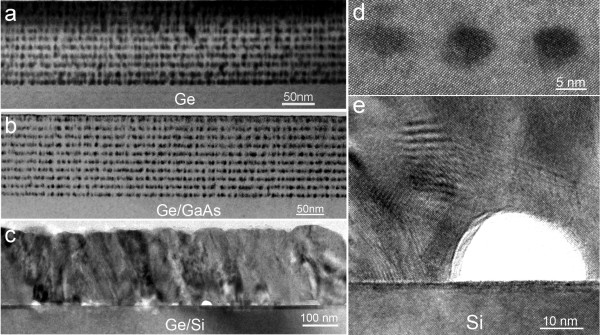
**Typical, high-resolution, and high-magnification TEM images of superlattices, nanodots, and interface**. Typical TEM images of GeMn/Ge superlattices grown on (**a**) Ge, (**b**) GaAs, and (**c**) Si." . (**d**) A high-resolution TEM image of GeMn nanodots in (a). (**e**) A high-magnification TEM image of GeMn/Si interface.

Figure 1a, b, c shows typical cross-sectional bright-field TEM images taken from samples S1 to S3 and shows GeMn/Ge superlattices grown on Ge, GaAs, and Si substrates under the same growth conditions, respectively. Ordered GeMn nanodot arrays are observed in Figure 1a, b, indicating that GeMn nanodot arrays can be formed both on Ge and GaAs substrates. However, no ordered nanodots were seen in the superlattice grown on Si substrates, as illustrated in Figure 1c. To understand the structural characteristics of grown nanodots, high-resolution TEM investigations were performed, and examples are shown in Figure 1d, e. As can be seen in Figure 1d, ordered and coherent GeMn nanodots can be clearly observed when they are grown on Ge or GaAs substrates. However, precipitates (moire fringe marked in Figure 1e) and lots of stacking faults appeared in the thin films grown on Si. Interestingly, a large number of voids can also be seen at the interface of the GeMn thin film and Si substrates. Since the lattice constant of Ge which is 0.5656 nm [17], close to that of GaAs (0.5653 nm), is larger than that of Si (with a lattice constant of 0.543 nm) [17], there should be almost no lattice mismatch occurring in the Ge/GaAs interface, but only a 4.35% lattice mismatch between the Si and Ge substrates. For this reason, the GeMn/Ge superlattices can be well epitaxially grown on both Ge and GaAs substrates, but for Si substrates, to release the strain induced by the lattice mismatch, the formation of stacking faults, voids, or precipitates will be hardly avoided [18]. It is also interesting to note that the nanodots grown on GaAs substrates seem better than those on Ge substrates. As those films were grown at the same condition, and GaAs has a nearly identical lattice constant as Ge, this phenomenon may be caused by other factors, such as different thermal coefficients for Ge and GaAs [19].

### The effect of Ge/GeMn thickness

**Figure 2:**
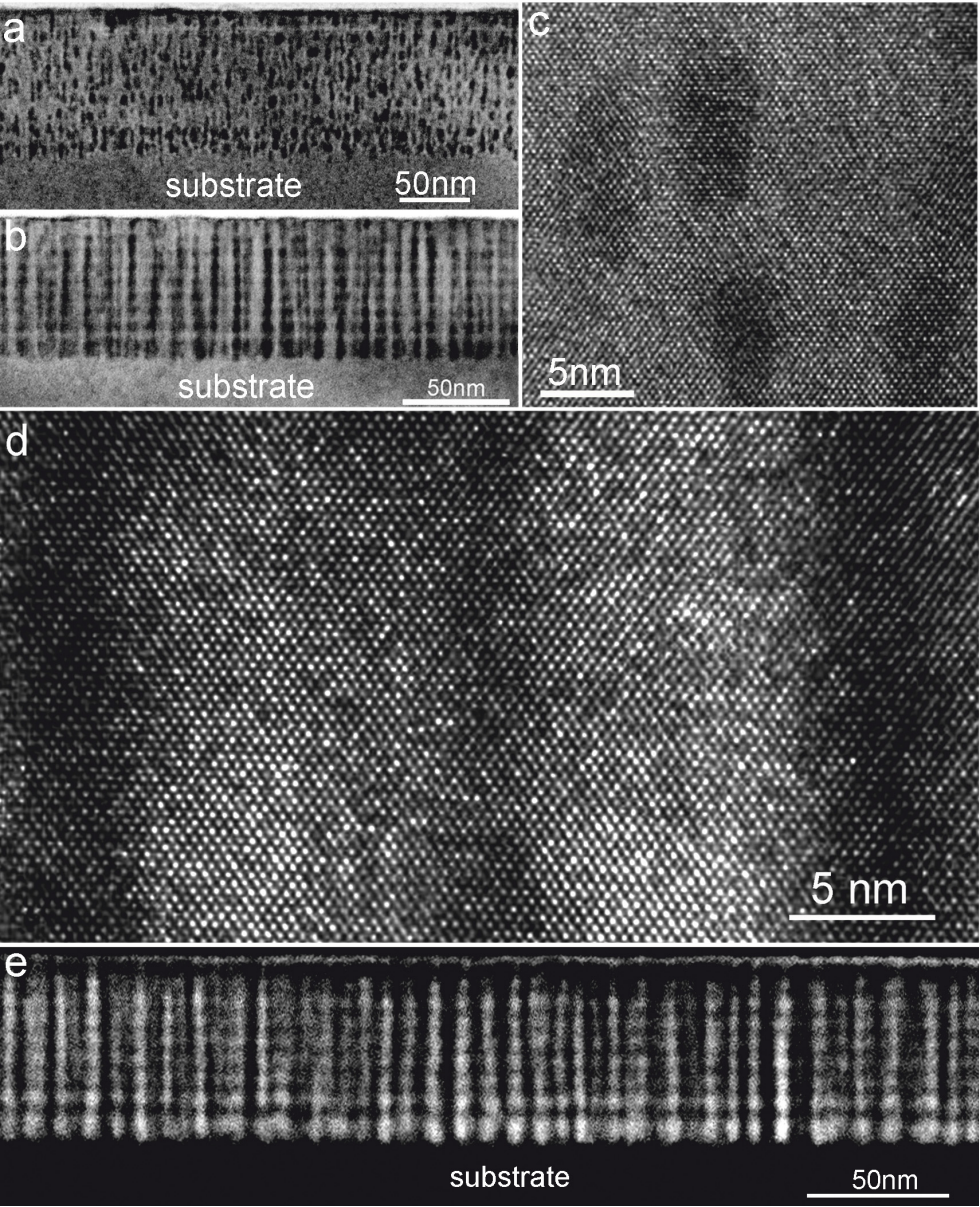
**Typical, high-resolution, and scanning TEM images of samples**. Typical TEM images of samples with different spacer thickness (**a**) S4 and (**b**) S5. High-resolution TEM images of samples (**c**) S4 and (**d**) S5. (**e**) A scanning TEM dark field image of sample S5.

It should be noted that coherent and ordered MnGe nanodot arrays require a critical growth window to ensure its reproducibility [16]. It is expected that a larger Ge spacer layer or a narrower MnGe layer would give rise to less strain coupling from the two adjacent MnGe layers, resulting in less ordered MnGe nanodots. In contrast, a thinner Ge spacer layer or a thicker MnGe layer (with more strain coupling) would cause vertically coalesced nanodots. Indeed, by decreasing the MnGe layer thickness to 1.2 nm while keeping other growth parameters identical, disordered MnGe nanostructures were observed (Figure 2a). On the other hand, when the Ge spacer layer was reduced to 4.6 nm, well-aligned Mn-rich nanocolumns with an Mn concentration up to 19% could be achieved (Figure 2b, e). Nevertheless, for both cases, coherent GeMn nanodots/nanocolumns can be observed, as displayed in the high-resolution TEM images in Figure 2c, d (for samples S4 and S5, respectively).

### The effect of Mn concentration

**Figure 3:**
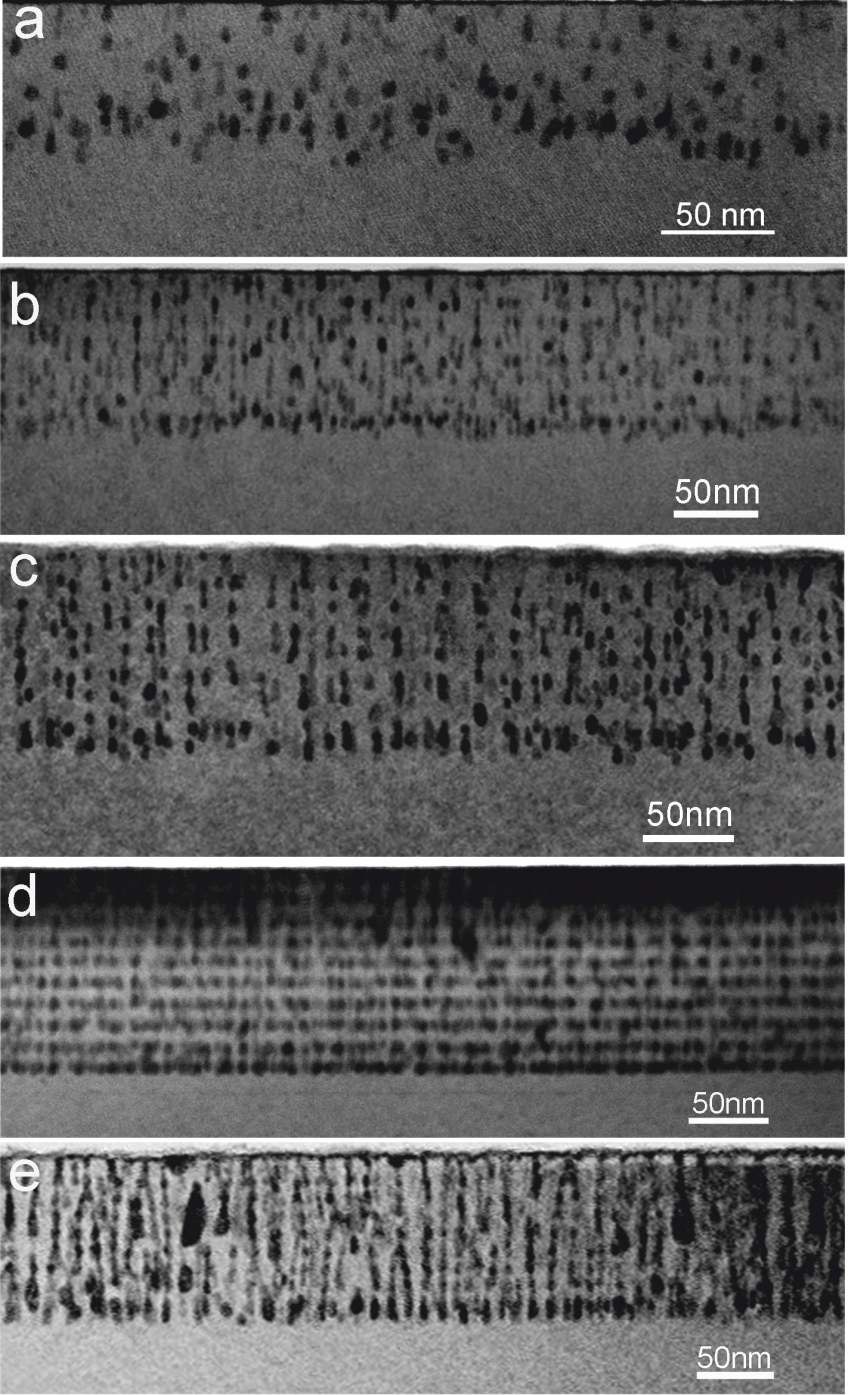
**Typical TEM images of samples with different Mn concentration**. Samples (**a**) S6, (**b**) S7, (**c**) S8, (**d**) S1, and (**e**) S9.

Other than the variation of the MnGe and Ge layer thicknesses, the change of Mn concentration can also be employed to control the behaviors of grown MnGe nanostructures. As shown in Figure 3, by varying the Mn concentration, the following sequence can be observed: disordered GeMn nanodots, ordered nanodots, and then ordered nanocolumns. Indeed, less Mn doped in Ge may not induce enough strain, which is critical to provide a nucleation site for the subsequent GeMn deposition. As a consequence, disordered GeMn nanodots are formed, as displayed in Figure 3a, b, c. On the other hand, by increasing the Mn concentration, the increased strain makes the two nearest vertical nanodots more easily merged and subsequently, the formation of nanocolumns (refer to Figure 3e). With an optimal Mn concentration between the two cases, the ordered GeMn nanodots can be formed without changing other growth parameters, as shown in Figure 3d.

### The effect of growth temperature

**Figure 4:**
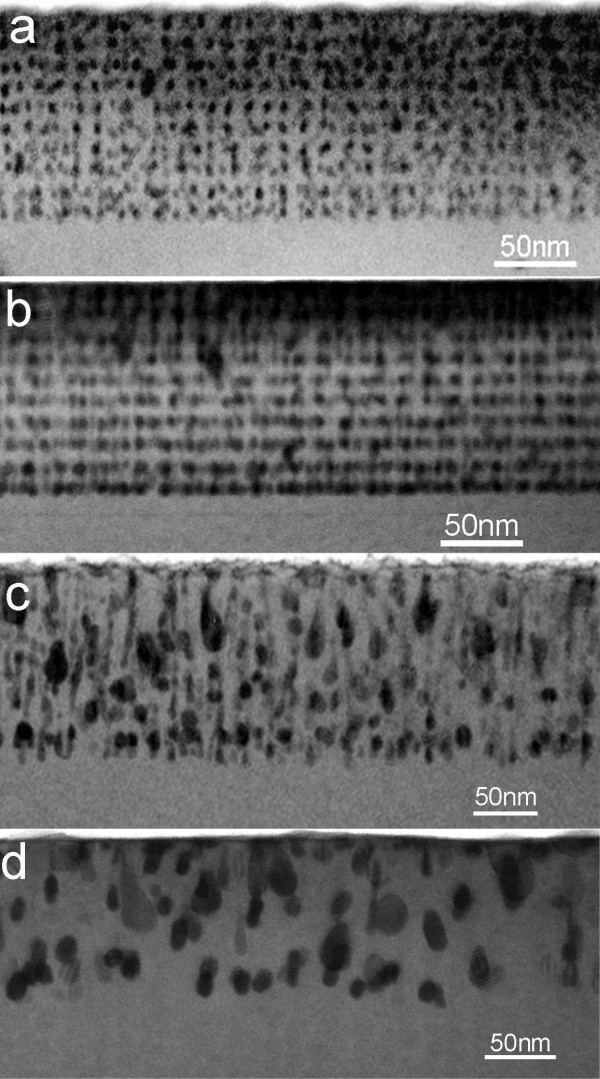
**The effect of the substrate temperature**. (**a**) S10 (27°C), (**b**) S1 (70°C), (**c**) S11 (110°C), and (**d**) S12 (150°C). The optimized growth temperature is found to be 70°C, as shown in (b).

Finally, the effect of the growth temperature on the MnGe nanostructures is investigated, and the results are shown in Figure 4. By comparing the morphology of Figure 4, the optimal temperature to secure the ordered and self-assembled nanodot arrays can be determined to be around 70°C. Since the Mn diffusion, promoting the formation of Mn-rich clusters, is closely related to the growth temperature, it is not active at lower growth temperatures, and less strain is induced, which would result in less ordered nanodots (refer to Figure 4a). However, higher growth temperatures cause the coalescence of the nearby nanodots and/or the possible formation of the secondary-phase Mn-rich clusters (for example Mn_5_Ge_3 _and Mn_11_Ge_8_) when the Mn diffusion is active, as shown in Figure 4c, d. For this reason, there should be an optimal growth temperature for the growth of ordered GeMn nanodot arrays.

According to our systematic study, the nanodot arrangement of the grown GeMn/Ge superlattices is sensitive to the growth conditions, such as substrate, Mn concentration, GeMn layer thickness, and growth temperature. There should be an optimal growth temperature and Mn concentration to secure the ordered nanodot arrays. Higher growth temperature and/or higher Mn concentration lead to the formation of Mn-rich secondary precipitates.

## Conclusions

In conclusion, we have studied the effect of substrate, GeMn/Ge thickness, Mn concentration, and growth temperature on the structure of the GeMn/Ge superlattices grown by MBE. We found that by varying the growth parameters, the structure of the GeMn/Ge superlattices can be changed from disordered GeMn nanodots to ordered GeMn nanodot arrays and then to well-aligned GeMn nanocolumns.

## Competing interests

The authors declare that they have no competing interests.

## Authors' contributions

YW and FX conceived the study. YW and ZML carried out the experiments and analysis. HX, XF, JD, KW, and JZ participated in the design of the study and contribute to the analysis. YW, JZ, and YW wrote the manuscript. All authors read and approved the final manuscript.

## References

[B1] Park YD Hanbicki AT Erwin SC Hellberg CS Sullivan JM Mattson JE Ambrose TF Wilson A Spanos G Jonker BT A group-IV ferromagnetic semiconductor: Mn_x_Ge_1-x_ Science 2002 295 651 654 10.1126/science.1066348 11809964

[B2] Park YD Wilson A Hanbicki AT Mattson JE Ambrose T Spanos G Jonker BT Magnetoresistance of Mn: Ge ferromagnetic nanoclusters in a diluted magnetic semiconductor matrix Appl Phys Lett 2001 78 2739 2741 10.1063/1.1369151

[B3] Bougeard D Ahlers S Trampert A Sircar N Abstreiter G Clustering in a precipitate-free GeMn magnetic semiconductor Phys Rev Lett 2006 97 237202 17280238 10.1103/PhysRevLett.97.237202

[B4] Bihler C Jaeger C Vallaitis T Gjukic M Brandt MS Pippel E Woltersdorf J Göseleet U Structural and magnetic properties of Mn_5_Ge_3 _clusters in a dilute magnetic germanium matrix Appl Phys Lett 2006 88 112506 10.1063/1.2185448

[B5] Biegger E Staheli L Fonin M Rudiger U Dedkov YS Intrinsic ferromagnetism versus phase segregation in Mn-doped Ge J Appl Phys 2007 101 103912 10.1063/1.2718276

[B6] Ahlers S Bougeard D Sircar N Abstreiter G Magnetic and structural properties of Ge_x_Mn_1-x _films: precipitation of intermetallic nanomagnets Phys Rev B 2006 74 214411

[B7] Xiu FX Wang Y Kim J Hong A Tang J Jacob AP Zou J Wang KL Electric-field-controlled ferromagnetism in high-Curie-temperature Mn_0.05_Ge_0.95 _quantum dots Nat Mater 2010 9 337 344 10.1038/nmat2716 20208524

[B8] Xiu FX Wang Y Wong K Zhou Y Kou X Zou J Wang KL MnGe magnetic nanocolumns and nanowells Nanotechnology 2010 21 5 10.1088/0957-4484/21/25/255602 20508314

[B9] Li AP Zeng C Van Benthem K Chisholm M Shen J Nageswara Rao S Dixit S Feldman L Petukhov A Foygel M Weitering H Dopant segregation and giant magnetoresistance in manganese-doped germanium Phys Rev B 2007 75 201201

[B10] Chen YX Yan SS Fang Y Tian YF Xiao SQ Liu GL Liu YH Meiet LM Magnetic and transport properties of homogeneous Mn_x_Ge_1-x _ferromagnetic semiconductor with high Mn concentration Appl Phys Lett 2007 90 052508 10.1063/1.2436710

[B11] Wang Y Xiu FX Zou J Wang KL Jacob AP Tadpole shaped Ge_0.96_Mn_0.04 _magnetic semiconductors grown on Si Appl Phys Lett 2010 96 3

[B12] Ayoub JP Favre L Berbezier I Ronda A Morresi L Pinto N Morphological and structural evolutions of diluted Ge_1-x_Mn_x _epitaxial films Appl Phys Lett 2007 91 141920 10.1063/1.2794723

[B13] Ottaviano L Passacantando M Picozzi S Continenza A Gunnella R Verna A Bihlmayer G Impellizzeri G Priolo F Phase separation and dilution in implanted Mn_x_Ge_1-x _alloys Appl Phys Lett 2006 88 061907 10.1063/1.2171485

[B14] Wang Y Xiu F Wang Y Xu H Li D Kou X Wang KL Jacob AP Zou J Effect of Mn concentration and growth temperature on nanostructures and magnetic properties of Ge_1-x_Mn_x _grown on Si J Crystal Growth 2010 312 3034 3039 10.1016/j.jcrysgro.2010.07.008

[B15] Wang Y Zou J Zhao ZM Direct structural evidences of Mn_11_Ge_8 _and Mn_5_Ge_2 _clusters in Ge_0.96_Mn_0.04 _thin films Appl Phys Lett 2008 92 101903 10.1063/1.2891069

[B16] Wang Y Xiu F Wang Y Zou J Beyermann WP Zhou Y Wang KL Coherent magnetic semiconductor nanodot arrays Nanoscale Res Lett 2011 6 134 21711627 10.1186/1556-276X-6-134 PMC3211181

[B17] Wang Y Zou J Zhao ZM Han XH Zhou XY Wang KL Mn behavior in Ge_0.96_Mn_0.04 _magnetic thin films grown on Si J Appl Phys 2008 103 3

[B18] Tan CS Reif R Theodore ND Pozder S Observation of interfacial void formation in bonded copper layers AIP 2005 87 201909

[B19] Wu HZ Fang XM Salas R McAlister D McCann PJ Molecular beam epitaxy growth of PbSe on BaF_2_-coated Si(111) and observation of the PbSe growth interface AVS 1999 17 1263 1266

